# Modeling of Molecular Interaction between Apoptin, BCR-Abl and CrkL - An Alternative Approach to Conventional Rational Drug Design

**DOI:** 10.1371/journal.pone.0028395

**Published:** 2012-01-10

**Authors:** Soumya Panigrahi, Jörg Stetefeld, Jaganmohan R. Jangamreddy, Soma Mandal, Sanat K. Mandal, Marek Los

**Affiliations:** 1 Department of Molecular Cardiology, Lerner Research Institute/NB-50, Cleveland, Ohio, United States of America; 2 Department of Chemistry, University of Manitoba, Winnipeg, Canada; 3 Manitoba Institute of Cell Biology, University of Manitoba, Winnipeg, Canada; 4 Faculty of Medicine, Memorial University of Newfoundland, St. John's, Newfoundland, Canada; 5 College of the North Atlantic, Clarenville, Newfoundland, Canada; 6 BioApplications Enterprises, Winnipeg, Manitoba, Canada; 7 Department of Clinical and Experimental Medicine (IKE) and Integrative Regenerative Medicine Center (IGEN), Linköping University, Linköping, Sweden; Roswell Park Cancer Institute, United States of America

## Abstract

In this study we have calculated a 3D structure of apoptin and through modeling and docking approaches, we show its interaction with Bcr-Abl oncoprotein and its downstream signaling components, following which we confirm some of the newly-found interactions by biochemical methods. Bcr-Abl oncoprotein is aberrantly expressed in chronic myelogenous leukaemia (CML). It has several distinct functional domains in addition to the Abl kinase domain. The SH3 and SH2 domains cooperatively play important roles in autoinhibiting its kinase activity. Adapter molecules such as Grb2 and CrkL interact with proline-rich region and activate multiple Bcr-Abl downstream signaling pathways that contribute to growth and survival. Therefore, the oncogenic effect of Bcr-Abl could be inhibited by the interaction of small molecules with these domains. Apoptin is a viral protein with well-documented cancer-selective cytotoxicity. Apoptin attributes such as SH2-like sequence similarity with CrkL SH2 domain, unique SH3 domain binding sequence, presence of proline-rich segments, and its nuclear affinity render the molecule capable of interaction with Bcr-Abl. Despite almost two decades of research, the mode of apoptin's action remains elusive because 3D structure of apoptin is unavailable. We performed *in silico* three-dimensional modeling of apoptin, molecular docking experiments between apoptin model and the known structure of Bcr-Abl, and the 3D structures of SH2 domains of CrkL and Bcr-Abl. We also biochemically validated some of the interactions that were first predicted *in silico*. This structure-property relationship of apoptin may help in unlocking its cancer-selective toxic properties. Moreover, such models will guide us in developing of a new class of potent apoptin-like molecules with greater selectivity and potency.

## Introduction

Aberrant expression of the *Bcr-Abl* oncogene is found with the frequency of ±95% of CML cases, and sporadically in other malignancies [1], [2]. Thus for therapeutic applications, especially for CML treatment, Bcr-Abl is an attractive target for rational drug design, although so far, only its tyrosine kinase domain has been utilized. Bcr-Abl oncoprotein, contains a number of distinct domains such as SH3, SH2, kinase domains, DNA binding domains, actin-binding domains, nuclear localization signals, nuclear export signal, and four proline-rich motifs that function as binding sites for the adaptor proteins such as, Grb2 and CrkL [2]. Several potent inhibitors have been developed and studied extensively [3]. Imatinib is currently widely used for the treatment of CML patients. Most chronic phase CML patients treated with imatinib as first-line therapy, initially maintain excellent response. However, failure of response occurs in advanced-stage CML patients due to drug resistance caused frequently by mutations within- or in the proximity to Bcr-Abl's ATP-binding pocket. Other types of changes have also been documented. For example, CML stem cells obtained from some patients with imatinib resistance, down-regulation the expression of the tumor suppressor PTEN could be detected [4]. Although, the second-generation tyrosine kinase inhibitors namely, dasatinib, nilotinib or bosutinib are effective on most of the Bcr-Abl mutations, some p-loop mutations or T315I substitution provide a very difficult resistance to most of the Bcr-Abl kinase inhibitors currently in use [2], [5], [6]. Other therapeutic strategies besides small molecule approach include the use of monoclonal antibodies [7].

Current understanding of resistance mechanisms include the ability of cancer cells to remove drugs (drug efflux) by the different transporters such as MDR1 (multidrug resistance protein 1) or P-glycoprotein, lack of bioavailability of drugs, and inhibition of transporter molecules such as SLC22A1 (solute carrier family 22 member 1), responsible for drug transport into the cell (drug influx), or mutations altering the interaction of drug with its target. These mechanisms however do not fully explain drug-resistance observed in all instances. One of the possible reasons could be the protein dynamics due to drug-protein binding (protein dynamics due to folding, change in shape and size). It is known that the kinase domain of Bcr-Abl is negatively regulated, in normal situation, by cooperative combination of the SH3 and SH2 domain by internally engaging the SH2 domain [8], [9]. Generally, SH3 domains serve as modules that mediate protein-protein associations along with SH2 domains and thus regulate cytoplasmic signaling. SH2 domains play important roles in (i) in cellular communication, (ii) in a variety signal transduction pathways, and (iii) in recognition of tyrosine-phosphorylated sites respectively. But inappropriate communication or misreading of the phosphorylated site could lead to undesirable activation of pathways [10], [11]. Negative regulation of Bcr-Abl by blocking these domains by apoptin-inspired small molecule to control its oncogenic role would be an attractive approach, as compared to conventional targeted drug design [12]. In this study, we present a possible alternative approach of inhibition of Bcr-Abl through surface interaction of SH3 domain by the apoptin molecule rather than binding to a narrowly-defined domain.

Apoptin has gained significant attention in recent years, both as a lead for the development of cancer-specific therapeutics, and also for its potential use as an indicator of cellular transformation processes. Apoptin is a 13.6 kD viral protein encoded by the *VP3* gene of Chicken Anemia Virus and is composed of 121 amino acids [13], [14]. It induces apoptosis independently of death receptor pathways in a broad range of transformed and cancer cells. Apoptin localizes in the nucleus in cancer cells, however in non-transformed or primary cells it is localized to the cytoplasm [15], [16], [17]. The cellular localization of apoptin is influenced by its phosphorylation status at theronine-108. Phosphorylated T-108 inhibits nearby nuclear export signal, thus leading to nuclear accumulation of apoptin [18], [19], [20]. Apoptin phosphorylation has been proposed to be regulated by Akt-activated CDK-2 and PKC kinase [21], [22], [23]. Thus, nuclear localization of apoptin and its interaction with specific signaling proteins plays a crucial role in its selective toxicity [19], [20], [24]. Highly organized recognition of specific target binding partners by signaling proteins is an important aspect of many cellular processes. The specificity of these interactions is determined by the physical, structural and chemical properties of the interacting proteins. Therefore, detailed knowledge about the 3D structures of the involved proteins is necessary to understand such interactions. Unfortunately, the 3D molecular structure of apoptin revealing its precise structure-function relationship has not yet been resolved by conventional crystallography or NMR studies. Determination of the 3D molecular structure requires that the molecule be in crystal form suitable for X-ray crystallographic study or to be of less than 30 kD for accurate NMR study. In case of apoptin, the 3D molecular structure could not be attained due to the inability of the molecule to be crystallized, and the nature of the molecule to stay in solution as globular multimers [25].

The alternative technique to study protein structures and to predict interactions with other proteins is ‘homology modeling’ and ‘virtual protein docking’ experiments. This novel method to identify the function of proteins, based directly on the sequence-to-structure-to-function paradigm, is broadly known as ‘computational protein modeling’ [26]. While employing this approach, it is possible to build the best-predicted structure of the protein from its amino acid sequences (target) on the basis of known 3D structure of related family members (templates). The ‘low resolution’ models obtained by homology modeling provide essential information of the spatial arrangement of important groups of residues.

Here we report for the first time, a simulated 3D model of apoptin generated by using homology modeling as a backup alternative to the conventional, crystallography- or NMR-based methods. We then use the calculated structural coordinates of apoptin to study its interaction with the 3D structure of CML-associated oncoprotein Bcr-Abl. Furthermore, we biochemically confirmed the accuracy of at least some elements of the model by showing that the modeled interaction between apoptin and Bcr-Abl indeed physically occurs between both proteins. We also examined the pathways and interacting network from a global perspective and experimentally validated some of the important molecules.

## Results

### Apoptin is toxic to both Bcr-Abl positive and -negative cells, and it inhibits Bcr-Abl phosphorylation/activation

**Figure 1:**
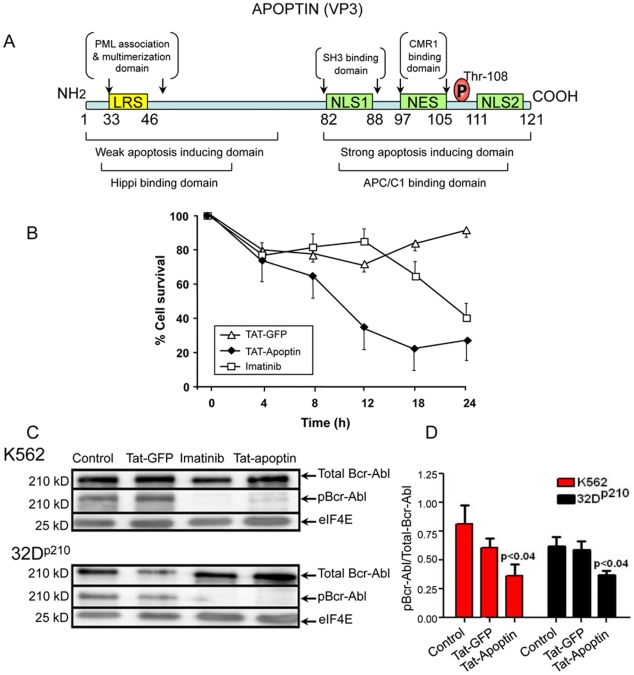
Schematic representation of the primary structure and functional domains of apoptin, its cytotoxic potency and inhibition of Bcr-Abl phosphorylation. (**A**) The SH3 binding domain is merged within NLS1 (amino acids, aa: 82–88). A pictorial representation of apoptin sequences (UniProtKB/Swiss-Prot entry P54094), LRS = Lecine-Rich Sequence, NLS = Nuclear-Localization Signal, NES = Nuclear Export Signal. (**B**) Cytotoxic activity of apoptin on Bcr-Abl positive 32D^p210^ cells: 32D^p210^ were grown in 96-well plates (10^4^ cells per well). Cells (in triplicates for each treatment) were treated with 1 µM Tat-apoptin, and Tat-GFP (negative control), or Imatinib for 0, 4, 8, 12, 18 and 24 h periods respectively. The percentage of viable cells, as assessed by MTT assay indicates that apoptin and Imatinib are both toxic to 32D^p210^ cells, and that apoptin's cytotoxic effect favorably compares to that of imatinib. Results are expressed as a percent of cell survival (mean ± SD). (**C**) Apoptin inhibits Bcr-Abl phosphorylation: K562 and 32D^p210^ cells were treated with 1 µM Tat-apoptin, Tat-GFP (negative control) or 1 µM imatinib (positive control). Cells were then harvested after 16 hrs and cell lysates were prepared. Representative Western blots show the expression levels of total and phosphorylated Bcr-Abl; equal loading was checked by the loading control, eIF4E. The upper panel of bands shows the expression of K562 cells and the lower panel shows the expression of 32D^p210^ cells. Lanes from the left: (1) no-treatment control cell, (2) Tat-GFP treated cell, (3) imatinib treated cell, and (4) Tat-apoptin treated cell respectively in both cell lines. (**D**) For quantitation, band intensities from immunoblots were scanned by Image Quant software (version 5.2, Molecular Dynamics®). During quantitation, the imatinib expression data was omitted in order to enable visualization of the apoptin effect with greater clarity. Bcr-Abl phosphorylation was significantly inhibited by apoptin. The quantitation data were normalized to the loading control (eIF4E) and expressed as a ratio of phosphorylated to the total Bcr-Abl and presented as mean ± SEM of three independent experiments.

The known domains of apoptin were designed and presented (Fig. 1A) to understand its cytotoxicity in relation to its various domains. As seen by the MTT assay performed on the representative murine 32D^p210^ cell line, Tat-apoptin efficiently killed those cells as compared to control (Tat-GFP, Fig. 1B). Furthermore, apoptin's toxicity favorably compared to imatinib. Evaluation of percentage cell survival was followed by the examination of the phosphorylation status of Bcr-Abl in the human leukemia cell line, K562 and the mouse cell line 32D^p210^. Tat-apoptin markedly inhibited phosphorylation of Bcr-Abl in both cell lines. Inhibition of phosphorylation of Bcr-Abl was evaluated by Western blotting (Fig. 1C) and quantified (Fig. 1D).

### Identification of the SH3 binding domain of apoptin

SH3 Hunter software, a web based server, was used to identify the SH3 binding domain of apoptin [27]. SH3- Hunter identified the sequences, 81 to 86 (PKPPSK) as the SH3 binding domain. SH3-binding domains have a consensus sequence:(-X-P-p-X-P-) with 1 and 4 being aliphatic amino acids, 2 and 5 always-, and 3 sometimes being proline (data not shown) [28].

### Apoptin colocalizes with nuclear phospho-Bcr-Abl and physically interacts with it

**Figure 2:**
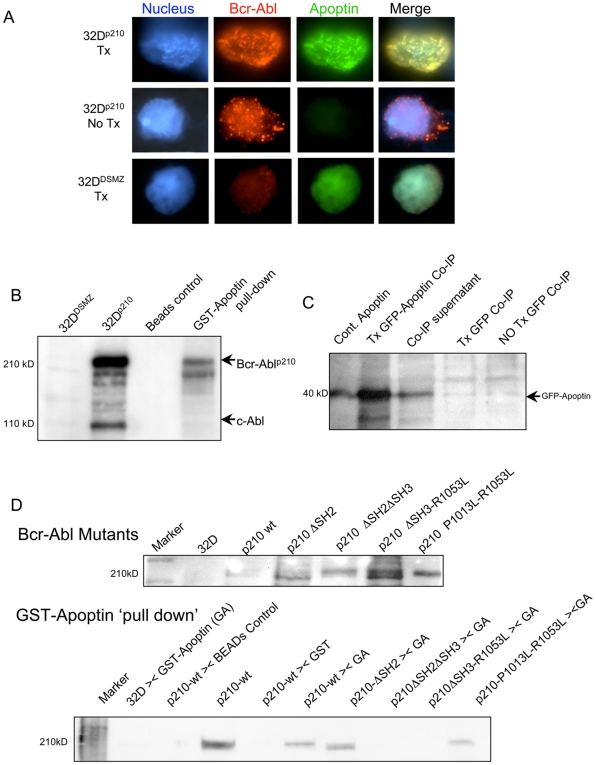
Interaction of apoptin with Abl and Bcr-Abl^p210^. (**A**) Indirect immunofluorescence showing the nuclear localization of Bcr-Abl. 32D^P210^ and 32D^DSMZ^ cells were transiently transfected with GFP-apoptin (green) and subjected to (immuno)fluorescence staining and detection. Apoptin localization was by GFP and Bcr-Abl was detected by staining with Bcr-Abl^p210^ with Cy3 tagged (red) secondary antibody. Nuclei were co-stained with DAPI (4, 6-diamidino-2-phenylindole: blue). Column 1 shows DAPI stained nuclei; columns 2 and 3 show the nuclear localization of Bcr-Abl and apoptin. Column 4 shows the merged image of nuclear co-localized of Bcr-Abl^p210^ and GFP-apoptin as small clusters (yellow). Abbreviations: Tx = cells transfected with GFP-apoptin, No Tx = no transfection with GFP-apoptin. (**B**) To demonstrate apoptin and Bcr-Abl interactions, 5–10 µg of GST-apoptin was used in the ‘pull-down assay’. The interaction was tested either on 500 µg of total cell lysates from Bcr-Abl expressing 32D^p210^ cells, or on Bcr-Abl non-expressing 32D^DMSZ^ cells. Lanes from the left: (1) the pull-down products of Bcr-Abl in 32D^DMSZ^ extracts (negative control), (2) 32D ^p210^ extract (positive control), (3) 32D ^p210^ extract treated with glutathione-sepharose beads (beads control), and (4) 32D^p210^ extract incubated with GST-Apoptin captured with glutathione-sepharose beads. (**C**) In order to detect apoptin and Bcr-Abl interaction by co-immunoprecipitation assay, 32D^p210^ cells were transiently transfected with GFP-apoptin (3 µg of pEGFP-apoptin plasmid for 2×10^6^ cells per transfection using lipofectamine transfection reagent) and cell lysates were incubated with anti-Bcr-Abl antibody followed by immunoprecipitation by protein G-sepharose beads; washed IP products were tested for the presence of apoptin (GFP-apoptin: 40 kDa) by immunoblot using anti-apoptin antibody. Lanes from the left: 1 - GST-Apoptin (positive control), 2 - GFP-apoptin Co-IP from transfected 32D^p210^ cells by anti-Bcr-Abl antibody, 3 - Co-IP supernatant/immunodepleted fraction from transfected 32D^p210^ cell lysates, 4 - 32D^p210^ transfected with GFP (Co-IP, negative control), and 5 - Co-IP from 32D^p210^ cells without transfection (Co-IP, negative control). (**D**) The Abl SH3 domain in Bcr-Abl^p210^ facilitates Bcr-Abl interaction with apoptin. 32D^DSMZ^ cells were transfected with various Bcr-Abl mutant constructs by lipofectamine using 3–4 µg purified plasmid DNA per 2×10^6^ cells. Specific mutant clones of transfected cells were selected by G418. Pull-down assays were performed 7–10 days following the selection and expressed proteins were detected by immunoblotting. The upper representative immunoblot shows various mutants of Bcr-Abl expressed in various 32D^DSMZ^ clones. The lower immunoblot shows results of GST-apoptin pull-down assay. The Src-homology domain mutant of Bcr-Abl^p210^ and GST-apoptin were used in this ‘pull-down’ (><) experiments using lysates from various Bcr-Abl mutant protein expressing 32D^DSMZ^ clones. The protein-protein complexes were analyzed for apoptin interaction by immunoblotting with rabbit anti-Bcr antibody. As seen (lane 6–9), the presence of an intact SH3 domain in the Bcr-Abl molecule is essential for its interaction with apoptin. Some degree of Bcr-Abl><apoptin interaction was also seen in the Abl-SH3 domain in Bcr-Abl, which was partially modified by single aa substitution (lane 10).

We performed immunofluorescent imaging studies to evaluate the subcellular localization of apoptin by comparison of the mouse myeloid cell line, 32D^p210^, stably transfected to express Bcr-Abl^p210^ with the Bcr-Abl non-expressing 32D^DSMZ^ cell line. In 32D^p210^ cells transfected with GFP-apoptin (Tx, 32D^p210^ and 32D^DSMZ^ cells transfected with GFP-apoptin), we observed the nuclear localization of apoptin detected with anti-GFP concomitant to the Bcr-Abl localization detected with Bcr-Abl^p210^ with Cy3 conjugated secondary antibody (column 2, 3, topmost panel, Fig. 2A). Colocalization of nuclear apoptin and phosphorylated Bcr-Abl was confirmed in the merged image (column 4, topmost panel, Fig. 2A). Column 1 in all three panels show DAPI stained nuclei.

Several well-characterized SH3 domains were previously identified as potential sites critical to ligand binding [29]. In preliminary experiments, we performed an array-based screening method (TransSignal SH3™ Domain Array1) to identify the interaction of apoptin with the SH3 domains of a known set of proteins (data not shown). This highly stringent SH3 domain interaction array screening indicated that apoptin strongly interacted with the SH3 domain of Abl. This preliminary observation was further substantiated by ‘pull-down assay’ and co-immunoprecipitation (Co-IP) studies using the Bcr-Abl^p210^ stably expressing 32D^p210^ cells and compared to the Bcr-Abl non-expressing 32D^DSMZ^ cells (Fig. 2B). For the GST-pull down assay recombinant GST and GST-conjugated apoptin were purified from IPTG stimulated transformed bacterial clones harboring the respective plasmids. The membrane was probed with anti-Bcr-Abl primary antibody. A representative blot from such an experiment shows the presence of Bcr-Abl (Lane 5) in the GST-Apoptin pull-down product (220 kD, Fig. 2B). This 220 kD protein ‘pulled down’ by apoptin, was confirmed by the presence of a similar band in the lysates of 32D^p210^ cells with stable expression of Bcr-Abl^p210^. This *in vitro* assay demonstrated apoptin interactions with Bcr-Abl. Non-specific interactions in the absence of GST-Apoptin (beads control, lane 4) were not detected. We further confirmed Bcr-Abl and apoptin interactions by Co-IP when GFP-Apoptin was transiently expressed in Bcr-Abl expressing 32D^p210^ cells (Fig. 2C).

### Apoptin interacts with Bcr-Abl via a specific motif

To identify the precise nature of apoptin and Bcr-Abl interaction in CML cells, we mapped the sites on apoptin responsible for interaction with specific region of Bcr-Abl^p210^. The murine bone marrow derived 32D^DMSZ^, 32D^p210^ cells and the human CML cell line K562 were grown in appropriate media and transfected with different apoptin mutant constructs (Materials and Methods, [18], [19]). The expression of these mutant derivatives of apoptin tagged with an N-terminal GFP was verified by SDS-PAGE and immunoblotting with mouse monoclonal anti-apoptin antibody. Apoptin was immunoprecipitated by murine anti-GFP antibody from lysates of transfected cells expressing various mutations of apoptin with murine anti-GFP monoclonal antibody and the protein complexes were analyzed to detect the presence of Bcr-Abl^p210^ by using rabbit monoclonal anti-Bcr antibody. Bcr-Abl^p210^ was found in the immunoprecipitates of full-length apoptin and apoptin derivatives that harbored amino acids from 74–100 (including the ‘Proline-rich sequence’: PRS), implying that this region of apoptin is important for interaction with Bcr-Abl^p210^ wt (data not shown). Interestingly, in this model system, the mutants Ala-108 and Glu-108 have a Thr-108 residue of apoptin replacement by alanine or glutamine respectively; these replacements render apoptin as non-phosphorylatable and are claimed by some authors to be non-toxic to cells [30]. Subsequently, the specific interactions between full-length GST-conjugated apoptin and Bcr-Abl^p210^ or various SH-domain mutant-constructs of Bcr-Abl expressed in 32D^DMSZ^ cells were studied (Fig. 2D). The mutants included: (*i*) Bcr-Abl^p210^ΔSH2: had an intact SH3, deleted SH2 and intact SH1, (*ii*) Bcr-Abl^p210^ΔSH2 ΔSH3: had a deleted SH2, deleted SH3 and intact SH1, (*iii*) Bcr-Abl^p210^ΔSH3-R1053L: had a deleted SH3, single amino acid (aa) substitution at SH2 and intact SH1, and (*iv*) Bcr-Abl^p210^P1013L-R1053L: had single aa substitution at the SH2 and SH3 domains respectively and intact SH1 domain. Mutants were selected according to their specific nature of SH-domain mutations to probe if apoptin interacted with the SH3 domain of Bcr-Abl. Full length Bcr-Abl, and its derivates with intact SH3-domain interacted with GST-Apoptin and were ‘pulled-down’ by glutathione sepharose beads, while other mutants lacking intact SH3 domain failed to show such interaction.

### Bioinformatics analysis of molecules known to interact with (Bcr-)Abl

**Figure 3:**
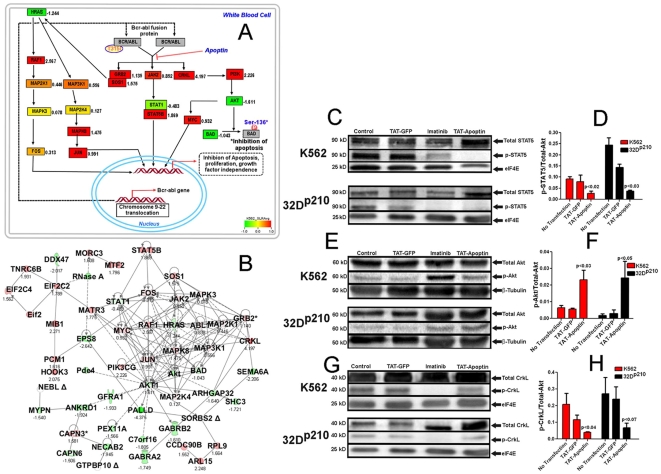
Visualization of pathways, interacting network, and validation of selected downstream regulators. (**A**) Bcr-Abl and its downstream effectors are shown by BioCarta pathways. Global gene expression data of K562 leukemia cells was taken from public database, analyzed, and visualized (GenMAPP). The expression values of signal log base e ratio (SLR) are shown outside the colored boxes. The software generated color codes denote up-regulation (dark-red) and down-regulation (blue-green). (**B**) Direct and indirect interacting network associated with Bcr-Abl was built utilizing global gene expression data and visualized by IPA. The up-regulated genes are shown in red and down-regulated genes are shown as green. The gene expression values (SLR) are also shown. (**C, E, G**) Apoptin induced inhibition of Bcr-Abl phosphorylation leads to the down-regulation of downstream regulators, STAT5, Akt, and CrkL respectively. K562 and 32D^p210^ cells were treated with 1 µM Tat-apoptin, Tat-GFP (negative control) and 1 µM imatinib (positive control) and cell lysates were prepared by harvesting cells after 16 h. Representative Western blots (divided into upper and lower panels representing the K562 cells and the second panel represents the 32D^p210^ cells) show the ratio of the expression levels of phosphorylated and total STAT5 (**D**), Akt (**F**), and CrkL (**H**) respectively. In all the immunoblots, lane 1 from no-treatment control cells, lane 2 is from Tat-GFP treated cells, lane 3 is from Tat-apoptin treated cells respectively for both cell lines. STAT5 phosphorylation was significantly inhibited by apoptin indicating that apoptin induced inhibition of Bcr-Abl phosphorylation decreases the activation of STAT5 through phosphorylation. On the other hand, Akt phosphorylation was higher, indicating that apoptin induced Akt activation, as previously published. For CrkL, apoptin induced inhibition of Bcr-Abl phosphorylation lead to the down-regulation of CrkL resulting in lower phosphorytion indicating that apoptin decreases the activation of CrkL, a down-stream substrate of Bcr-Abl. For quantitation, band intensities were scanned by Image Quant software (version 5.2, Molecular Dynamics®). During quantitation, imatinib expression data was omitted in order to enable greater visualization of the apoptin effect. The quantitation data were normalized to the loading control (eIF4E/β-tubulin) and expressed as a ratio of phosphorylated to the total protein and presented as mean ± SEM of three independent experiments.

Global gene expression data for K562 cells was analyzed as published [31]. Pathway analysis and visualization was performed using the GenMapp and pathVisio bioinformatics tool [32], [33]. The BioCarta pathway for the Bcr-Abl regulated genes in K562 cells was analyzed and visualized using PathViSio (Fig. 3A). Another bioinformatics tool, ‘Ingenuity Pathways Analysis’ was used to build and to identify the directly- or indirect interacting network of molecules (Fig. 3B) [34]. Bioinformatics analyses were validated experimentally for some of the molecules present in the pathways and in the networks.

### Apoptin down-regulates the Bcr-Abl kinase activity and modulates the phosphorylation of downstream kinases

To study the down-stream effects of apoptin and Bcr-Abl interaction, we examined the expression and phosphorylation status of Bcr-Abl^p210^ and other major down-stream Bcr-Abl targets like STAT5, CrkL, c-Myc and Akt in mouse (32D^p210^) and human (K562) CML cell lines. In immunoblotting experiments, we measured the phosphorylated and total proteins. The overall results indicate that apoptin induced inhibition of Bcr-Abl (auto)phosphorylation (Fig. 1C), down-regulated STAT5 (Fig. 3CD) and CrkL (Fig. 3GH) and activated Akt (Fig. 3EF). These results are consistent with global gene expression pattern observed in untreated K562 cells (Fig. 3AB). In all experiments, a comparison was done with imatinib treated cells (positive control), a known Bcr-Abl inhibitor and clinically-used CML-therapeutic. We quantified the relative phophorylation level of Bcr-Abl^p210^ by immunoblotting with phospho-Bcr-Abl-specific antibodies and thus we could assess the inhibition of activated Bcr-Abl by apoptin, which was highly significant (p<0.01, 0.04) in both cell lines.

STAT kinases serve a dual role of signal transducers and activators of transcription. Among the large family of over 30 STAT proteins, STAT5 has been identified as a key factor involved in anti-apoptotic signaling and malignant transformation in CML. Here we show that STAT5 phosphorylation was markedly reduced in K562 cells following Tat-apoptin treatment, which was comparable to imatinib (Fig. 3CD). Similar statistically significant results (p<0.03) were obtained in Bcr-Abl^p210^ expressing mouse cell line 32D^p210^ (Fig. 3CD). Moreover, we also studied the downstream consequences of Bcr-Abl inhibition by apoptin on the phosphorylation status of CrkL. This 39 kD protein is involved in β-integrin signaling and is a prominent substrate for activated Bcr-Abl kinase [35]. We observed a significant (p<0.04) inhibition of CrkL phosphorylation in K562 cells treated with 1 µM Tat-apoptin for 16 hrs comparable to imatinib treated cells (quantification not shown for imatinib) (Fig. 3GH). Marked inhibition of CrkL phosphorylation was also in Bcr-Abl^p210^ expressing 32D^p210^ (Fig. 3GH). For analysis of the pro-apoptotic effect of apoptin in Bcr-Abl expressing cells, we examined its effect on the signaling protein Akt and compared it to imatinib (Fig. 3EF). Interestingly, although Akt is known mediator of cell survival, we observed a marked augmentation of Akt phosphorylation 16 h after either apoptin or imatinib treatment of K562 and 32D^p210^ cells (Fig. 3EF). It is possible that the activated Akt may still act in an anti-apoptotic manner in Bcr-Abl expressing cells if re-located to the nucleus as previously proposed [23], [36], [37]. Our results were consistent with the global gene expression data for STAT5, CrkL, and Akt (Fig. 3AB).

### Development of homology based three-dimensional model of apoptin

**Figure 4:**
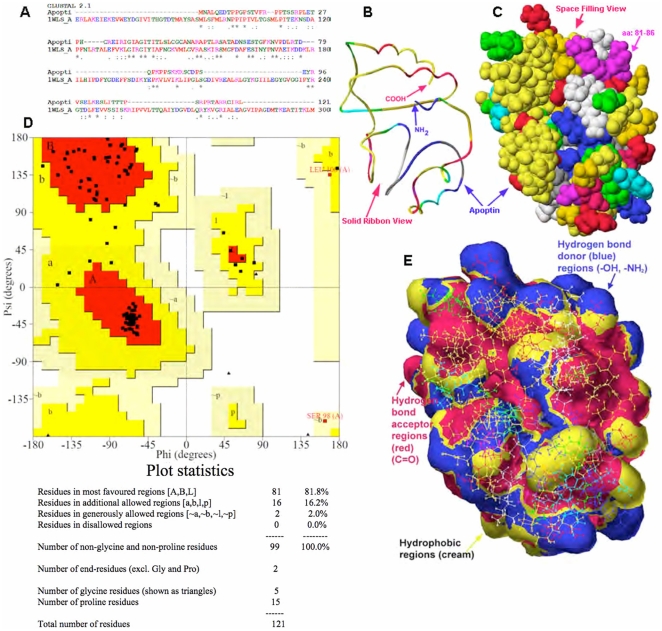
Sequence alignment and 3D model of Apoptin (aa: 1–121). (**A**) A representative sequence alignment between apoptin residues and the residues of one of the templates from a group of templates (Table1) is shown. (**B**) Solid ribbon view of full-length (aa: 1–121) of 3D model for apoptin and its amino and carboxyl terminals are shown. (**C**) Space filling view of apoptin model, showing the potential hydrophobic proline rich interacting area (PKPPSK, aa: 81–86, pink colored region, top right) is shown. (**D**) Ramachandran plot showing the N-Cα and Cα-C bonds in the apoptin polypeptide chain represented by the torsion angles phi (φ) and psi (ψ); quality of the model was examined by this plot (all atoms are within the allowed regions) and by the G-factors values (the overall value for G-factors is −0.35). (**E**) Solvent accessible surface area shows the regions of hydrophobic (large cream colored region at the surface) where protein-protein interactions could occur and the hydrophilic regions that are involved in hydrogen bonding, hydrogen bond acceptors (red color) and hydrogen bond donors (blue color). Additional information on apoptin structure could be found in Coordinates S1, S2, S3.

In this part of the study, unknown 3D structure of apoptin was approximated by a comparative- or homology protein modeling. To this end, we used the protein sequence (target) based on the known 3D structure of proteins with domains that have related peptide sequences (Table 1). The 3D structures of several known templates (Table 1) with identified partial homology to apoptin were used to build the 3D structure. To build the apoptin model, we used several modeling programs such as Modeller [38], [39] and DeepView [40], as well as project mode and the alignment mode of Swiss Model web based server [40]. After numerous trials using different templates, we were able to build the full-length apoptin model. It is worth mentioning that model quality was different when we used different chain of the same structure as a template. To understand this difference in model quality, we superimposed two chains in one of the templates (for example: PDB code: 1WLS, chains A and B) and noticed the differences between two chains as jugged by the RMS value (0.877 Å) when two chains were superimposed. The difference in the RMS value may be due to the missing residues in some cases and/or due to differences in resolution between two chains. The Swiss Server Alignment mode provided better results when multi-sequences were used. The T-Coffee or ClustalW2 multiple sequence alignment tools [41] were used to align a group of five or six sequences from a group of templates (Table 1). Modeller [38], [39] provided the best results. One of the best models was used for further studies (Fig. 4BC). The coordinates of this model are submitted as supporting materials (Coordinates S1, S2, S3).

**Table 1:** List of template proteins used to build apoptin model.

PDB codes*	PROTEINS
1WLS_A	L-asparaginase I homologue from Archacea (*pyrococcus horikoshii*)
1OQY_A	Human UV excision repair protein RAD23 homolog A
1Q9J_A	Ml2640c from mycobacterium leprae
Apoptin	PapA5, a phthiocerol dimycocerosyl transferase from Mycobacterium tuberculosis.
1E3I_A	Murine alcohol dehydrogenase, class II
1E3L_A	P47H mutant murine alcohol dehydrogenase, class II
2GYZ_A	Neurotrophic factor artemin, isoform 3
2GYR_A	Neurotrophic factor artemin, isoform 3
2GH0_C	DNA-directed RNA polymerase alpha chain
1QZE_A	UV excision repair protein RAD23 homolog A
1WNF_A	PH0066 (L-asparaginase) from Archacea
2ASK_A	Human artemin
2UYQ_A	A viral protein encoded by the *VP3* gene of Chicken Anemia Virus

*Data source: Protein Data Bank (RCSB-PDB).

Subsequently, a Ramachandran plot was performed to verify the quality of the model (Fig. 4D) [40], [42], [43], [44]. The N-Cα and Cα-C bonds in a polypeptide chain are relatively free to rotate. These rotations are represented in the plot by the torsion angles phi (φ) and psi (ψ), respectively. The structure was examined for close contacts between atoms for each of these conformations. Atoms were treated as hard spheres with dimensions corresponding to their van der Waals radii. Therefore, angles that cause spheres to collide correspond to sterically disallowed conformations of the polypeptide backbone. Disallowed regions involve steric hindrance between the side chain methylene group and main chain atoms. This model of apoptin (aa:1–121), most residues, about 81.8% of the residues (81 residues), were in the most favored regions, 16.2% in the additional allowed regions, 2.0% in the generously allowed regions, and no residues were fall in the disallowed regions according to Ramachandran plot (Fig. 4D). According to Procheck, the overall G-factor was about −0.35. After examining the accuracy of the model, all atoms of the molecule were locked, hydrogen atoms were added and molecular mechanics (MM2) and molecular dynamic simulations was performed at 1000 K for 50 ps simulation duration with 0.001 simulation time-step (ps). All theoretical calculations and visualization were performed using ‘Scigress Explorer Ultra’ associated with the ‘Gaussian03’ software [42], [45], [46].

This model was used to examine solvent accessible surface area (Fig. 4E) to identify the surface (large patches, cream color, of hydrophobic areas) of the protein that are involved in interactions with other proteins and hydrophilic regions that involved in hydrogen bonding, hydrogen bond acceptors (red color) and hydrogen bond donors (blue color). This model was further used to perform virtual docking experiments to examine and to understand the interactions between apoptin and Bcr-Abl.

### Virtual docking of Bcr-Abl and apoptin model

**Figure 5:**
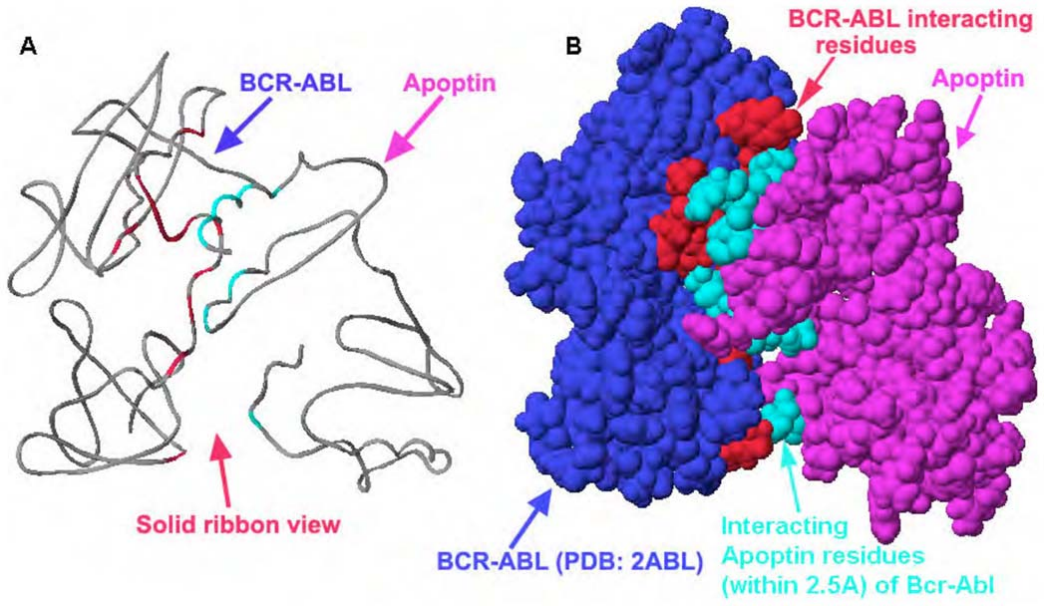
Modeled interactions between apoptin and Bcr-Abl. (**A**) Shows the interaction between apoptin and the SH3-domain of Bcr-Abl (solid ribbon view, showing the two terminals of two proteins) obtained by performing virtual docking experiment between apoptin model and the X-ray structure of Bcr-Abl-SH3 domain (PDB: 2ABL). (**B**) Shows the space filling docking view of the interactions between apoptin (pink) and the SH3-domain of Bcr-Abl (blue), the 13 residues (red) of Bcr-Abl and 13 residues (light blue) of apoptin that are within 2.5 Å to each other; some of the proline-rich (PxxP) SH3-binding residues (Table 2) are present and at least five direct hydrogen bonding are possible in between them (Table 3). Additional information on apoptin interaction with BcrAbl could be found in Coordinates S4, S5, S6.

To examine protein-protein interaction between apoptin model and the 3D structure (PDB code: 2ABL) of Bcr-Abl, molecular docking experiments were performed using ClusPro [45], [47] and Hex [48] web based protein docking servers. The ClusPro provided about ten structures. One of the lowest energy structures (Fig. 5AB) was used for further analysis. All atoms are locked and hydrogen atoms were added and energy optimization was performed. Finally, interacting residues between two molecules that are within 2.5 Å of each other were identified and given in the Table 2 and in Table 3 corresponding hydrogen bond distances are presented.

**Table 2:** Interacting amino acid residues of apoptin and Bcr-Abl.

Apoptin Interacting residue	BCR-ABL residue
Thr8	Lys29
**Lys82** *	**Glu68**
**Pro83** *	**Ser66, Glu68**
**Lys86** *	**Pro63**
**Lys87** *	**THR62**
**Thr108**	**Ser71**
Arg111	His74
Pro112	His74
Thr114	Tyr84, Leu85
Ala115	Val77
Lys116	His74
Arg118	Ser 78, Glu100
Ile119	Pro76

*The SH3 interacting amino acids in the proline rich PxxP region of apoptin are marked as bold.

**Table 3:** Amino acid residues forming hydrogen bond between apoptin and Bcr-Abl.

Apoptin residue	BCR-ABL	Hydrogen Bond distance (Å)
Thr8	Lys29	1.983
**LYS82** *	**Glu68**	**1.86**
**Lys86** *	**Glu98**	**2.072**
Lys116	His74	1.912
Arg118	Glu100	2.102

*Hydrogen bonding forming residues between the Bcr-Abl and the proline rich PxxP region of apoptin are shown in **bold**.

### Shape and sequence similarity of apoptin and the SH2 domain of CrkL

**Figure 6:**
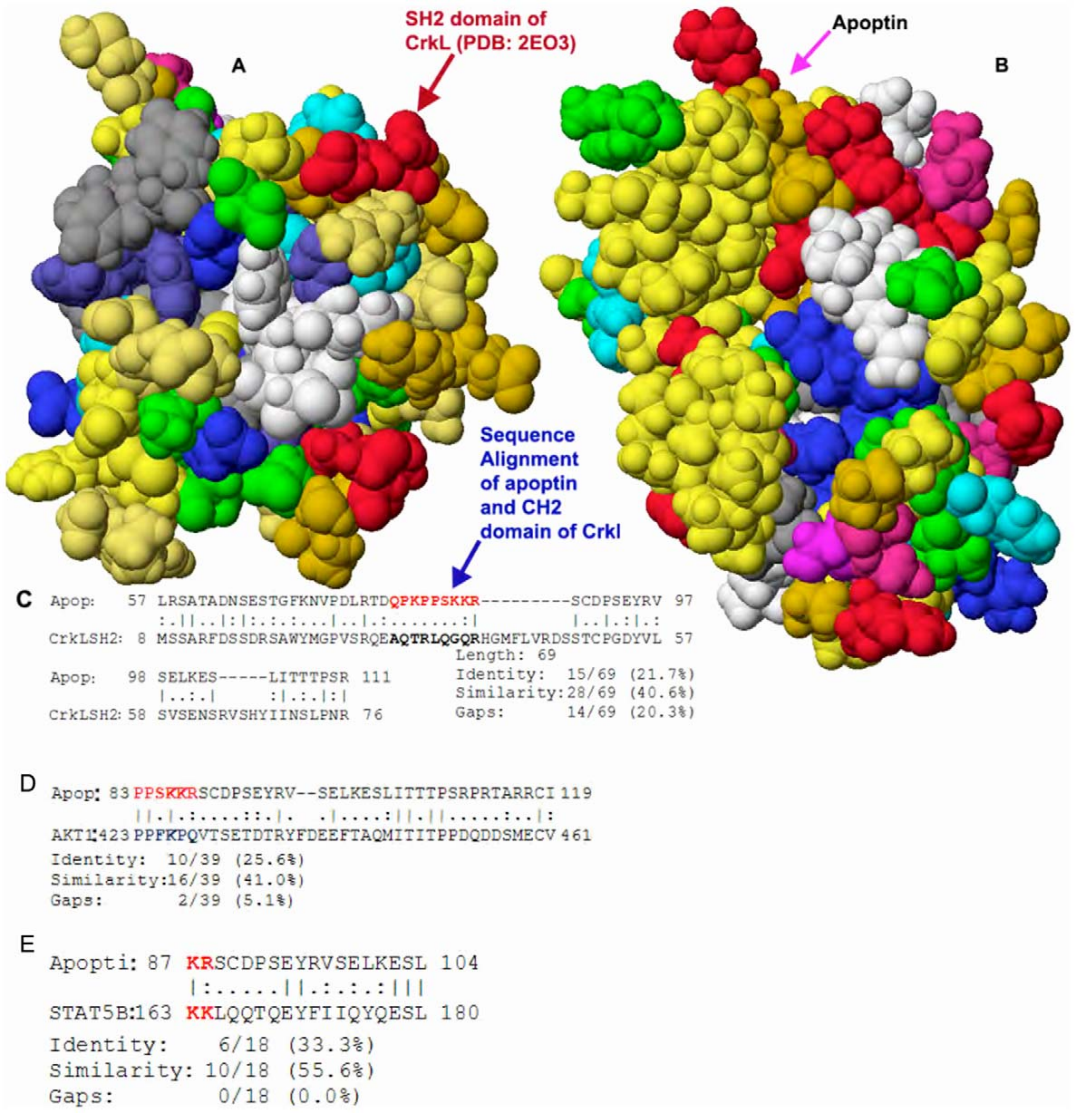
Sequence alignment and interactions between apoptin and adopter proteins CrkL, Akt1 and STAT5. Space filling views and similarity in sequences between apoptin and the SH2-domain of the adopter protein CrkL (**A**), apoptin's SH3-binding domain residues (**B**), 81 to 86 (PKPPSK), are within the SH2-domain of CrkL (**C**). In addition, the similarity in sequences between apoptin and the adopter proteins Akt1 (**D**) and STAT5 (**E**) suggesting that apoptin might directly interact in the CrkL, Akt1 and STAT5 interacting sites in addition to the SH3-binding domain of Bcr-Abl and could block further propagation of survival and proliferation signaling.

CrkL domains were identified using Prosite [49], a web based server. Prosite identified SH2 (aa 14–102) and SH3 (aa: 123–183) domains of CrkL. Sequence alignment of apoptin and the SH2 domain of CrkL were performed. Interestingly, we observed that the sequence of apoptin was somewhat similar (identical residues 21.7%, and similarity 40.6%) to that of SH2 domain of CrkL, and apoptin's proline-rich segment (aa: 81–88) was found to be within this aligned region of SH2 domain. We then compared the shape of known 3D structure (PDB code: 2EO3) of SH2 domain of CrkL and apoptin model. Sequence alignment structural similarities are shown in figure 6A, B, and C respectively. We also performed the virtual docking experiments between the structure of SH2 domain of CrkL and the structure of Bcr-Abl (PDB code: 2ABL).

## Discussion

The 3D structure of apoptin has been unknown due to numerous reasons (lack of a suitable crystals, multimerisation in solution), furthermore apoptin modeling is challenging due to the low number of suitable templates. We have been able to build a model of apoptin by applying a comparative or homology protein modeling approach despite low identity (about 31%) and similarity (about 52%) of the templates. Figure 4A–C, and E shows the sequence alignment of the templates, ribbon view, space filling full-length model of apoptin, and Ramachandran plot, and solvent accessible surface area respectively. This model was used to virtually examine various binding interactions with Bcr-Abl by performing virtual docking experiment between apoptin and the X-ray crystal structure of Bcr-Abl (PDB code: 2ABL). First, accessible surface area for apoptin was identified. As shown in figure 4E, the large cream colored area is the hydrophobic region, the sites for protein-interaction, purple-red areas and blue areas are hydrophilic regions, purple-red indicates hydrogen bonding acceptors (for example, C = O) and blue regions indicate hydrogen bond donors (for example, N-H or O-H).

Using this model, we have been able to identify the nature of interactions and hydrogen bonding between the residues of SH3 domain of Bcr-Abl and apoptin (Table 3). Subsequently, we have experimentally verified the observed interaction between apoptin and the SH3 domain of Bcr-Abl oncoprotein. In this model system, 13 aa (Fig. 5B, red) of SH3 domain of Bcr-Abl are approximated within 2.5 Å of apoptin and 13 aa (Fig. 5B, light-green) of apoptin are within 2.5 Å of Bcr-Abl residues. Interestingly, some of the proline rich PxxP sequences (aa: 81–86, QPKPPSKKR) (Fig. 5AB) are involved in these interaction among other nearby residues and at least five pairs of direct hydrogen bonding are possible between them (Table 2). This low-resolution model provides information about the interaction between Bcr-Abl and apoptin and it helps us to explain the probable mode of action; moreover, our experimental pull-down assay' and co-immunoprecipitation studies confirm occurrence of those interactions in cell nuclei.

We not only show for the first time, that Tat-apoptin, a cell-penetrating conjugate of apoptin strongly binds to the SH3 domain of Bcr-Abl, but also it modifies the phosphorylation status and thus the activity of Bcr-Abl, and several of its downstream targets. These changes lead to the anti-proliferative effect and induction of intrinsic apoptotic pathways in rapidly dividing CML cells. Using human CML cell line, K562 and Bcr-Abl^p210^ expressing murine cell line 32D^p210^ as models, we observed that these cells are significantly responsive to apoptin. These highly proliferating human and murine cell lines have a high cytoplasmic Bcr-Abl^p210^ pool and thus the cell culture condition mimic the blast crisis stage of CML. Furthermore, as in CML, the central mitogenic Ras-MAPK cascade is also activated, similarly as in our model cell lines. Our findings corroborate well with previous studies, by Kardinal and colleagues, involving a similar approach directed towards the Grb2-SoS-Ras-MAP kinase (Erk) pathway [50]. In these experiments, small, high affinity peptides blocking the N-terminal SH3 domain of Grb2 were applied. Their results indicate that peptide based inhibitor of Bcr-Abl kinase or its down-stream targets could be valuable anti-CML tool if combined with conventional cytotoxic therapy [50]. We have also observed that apoptin-derived peptides capable of interaction with SH3 domain are toxic against Bcr-Abl expressing cells (data not shown). We have further demonstrated that apoptin, unlike Imatinib/Gleevec, was effective both against Bcr-Abl positive and also Bcl-Abl negative cells. We thus hypothesize that apoptin-based therapeutics would be not only more effective, but have the additional advantage that they would be less prone to the development of resistance.

Activation of Bcr-Abl is critical for the development of CML. Different downstream molecules and pathways such as the Grb2-Ras-Raf-Mek1/2 Erk pathway, the PI3 kinase pathway involving Gab2 [51], [52], [53], [54], [55], the Jak2-STAT3 pathway [56], [57], and the Bcr-Abl-STAT5 pathway [58], [59] are implicated as shown in figure 3A. Using bioinformatics approaches, we visualize the relationship between these component molecules and pathways using global gene expression data. A comprehensive analysis of molecular interactions of Bcr-Abl target molecules that are either directly (solid lines or solid lines with arrows) or indirectly (broken lines or broken lines with arrows) involved are shown in an interaction network (Fig. 3B). Interrelationship between molecules is clearly visualized their activated (pink color) or repressive (green color) states. In this figure, expression values are also shown. As shown in diagram 3A, the network of genes and proteins is very complex and in the context of drug design it is essential to consider these interrelationships to avoid drug toxicity.

In our previous studies, we have shown that direct apoptin-Akt interaction initiates nuclear trafficking of Akt. Interestingly, nuclear Akt, instead of activating an anti-apoptotic response initiates apoptosis by a process that is only partially understood [36]. Our observation corroborates well with recent data from other studies, showing that Akt inhibitors have been only moderately successful in experimental cancer therapy [60]. Furthermore, similar to the earlier observed nuclear transfer of Akt, we have also observed the nuclear transport of apoptin-interacting protein Bcr-Abl. We hypothesize that nuclear re-location of Bcr-Abl may markedly affect its biologic properties.

The adaptor proteins CrkL forms a complex with Bcr-Abl leading to Bcr-Abl–dependent phosphorylation of CrkL and subsequent phosphorylation of c-Cbl may contribute to Bcr-Abl dependent activation of PI3 kinase [61]. Multiple downstream targets of PI3 kinase have been identified. But apoptin interaction might block complex formation between the adapter protein CrkL and Bcr-Abl. Nuclear localization of apoptin is essential to block the complex formation between Bcr-Abl and CrkL. We have examined the nuclear localization of apoptin as shown Figure 2A. As previously mentioned, apoptin is a cytoplasmic molecule but nuclear localization occurs upon phosphorylation at Thr108 in transformed cells, thus in CML-cells it is predominantly nuclear. These interactions and trans-activation of CrkL and c-Crk II by activated Bcr-Abl kinase and their functional consequences are well documented [35], [62]. In the current study, we demonstrated for the first time that apoptin inhibits phosphorylation of CrkL in Bcr-Abl expressing cells. This observation indicates that apoptin can indirectly affect growth-supportive role of phosphorylated CrkL by inhibiting Bcr-Abl kinase. Overall, these observations signify apoptin as a negative-modulator of Bcr-Abl kinase activity, and indirectly, of the multiple cell proliferation and anti-apoptotic pathways that are fuelled by Bcr-Abl. We also consistently observed the activation of Akt upon apoptin and/or imatinib treatment in Bcr-Abl expressing cells. Akt is a downstream target for Bcr-Abl kinase and known to interact with apoptin. However it also functions independently of Bcr-Abl, for example, it is one of the key effectors of the PI3-K/PDK1-2 pathway upon cell membrane triggering. Beside its pro-survival function, activated Akt if located in the nucleus, will promote cell death rather than cell survival [23], [36], [37].

STATs act as regulators of cell proliferation [63]. The N-terminal regulatory region of Abl protein contains the SH2 and SH3 domains which are important for the regulation of activity *in vivo* [64]. We have previously reported that apoptin productively interacts with the SH3 domain of p85 regulatory subunit of PI3-K [18]. In the current study, we report for the first time that apoptin inhibits STAT5 activation in Bcr-Abl expressing cell lines. Furthermore, since STAT5 also regulates the Bfl-1 family gene A1 that reportedly collaborates with *c-myc* and is required for Bcr-Abl transformation [65], this observation strongly supports the role of apoptin as a proliferation inhibitor of CML cells.

Different components of the Bcr-Abl downstream pathways are involved in the pathogenesis of CML and are highly active compared to normal cells. Hyperactivation of STATs, Ras-MAPK or CrkL-integrin pathways lead to the development of characteristic CML pathologic features. Apoptin affects many of these signaling events, and thus it is well suited for targeting the cellular signaling environment of Bcr-Abl expressing cancer cells. In addition, apoptin is a good candidate to serve as a model/lead molecule for the development of smaller peptides or peptidomimetics that would target multiple cell proliferation and anti-apoptotic pathways. This may be of advantage also for CML-treatment because advanced highly mutated CML-cells may no longer solely rely on Bcr-Abl as the driver of cell proliferation (hence, acquired resistance to Imatinib). Apoptin acts on multiple targets related to cell proliferation by blocking their association with Bcr-Abl rather than binding. Thus, this is an alternative approach to the conventional target based rational drug design to block the interactions of adapter molecules rather than binding a small molecule to the active site. When a small molecule diffuses into a macromolecule, it alters the shape and size of the macromolecule leading to its conformation change. These changes in shape, size, and dynamics could lead to the activation/deactivation of undesired bio-molecules that in turn may yield detrimental effects.

To improve the potency and specificity of small apoptin-like peptides, designing new small molecules with proper shape, number of proline residues in appropriate positions, and capability of nuclear trafficking is essential. To avoid undesirable drug effects, repetitive evaluation of potency, examination of global gene expression, and extensive bioinformatics analysis are indispensable until a drug-like molecule with desired properties is achieved. Present study, model-structure-function relationship, provides such opportunities to design next generation of apoptin-like molecules with desired properties. We are aware of limitations of computational modeling of protein structure. However since we are able to confirm by biochemical methods the predicted intermolecular interactions, we are convinced that the provided model is highly accurate.

## Materials and Methods

### Three-dimensional/3D modeling

The homology modeling approach was used to generate 3D structures of apoptin, a viral protein encoded by the *VP3* gene of Chicken Anemia Virus that is composed of 121 amino acids (13.6 kDa). The crystal structure coordinates of the PDB id 1WLS, L-asparaginase from the hyper-thermophilic archaeon *Pyrococcus horikoshii* was used as one of the templates. The sequence of apoptin has about 31% identity and about 52% similarity with the sequence of the PDB id 1WLS. As mentioned earlier, different approaches were used to build the apoptin model. For alignment mode (Swiss Model web based server), five sequences, including apoptin, with known 3D structures were aligned using T-Coffee Multiple Sequence Alignment Tool [41] and then submitted for model building. For project mode (Swiss Model web based server), the DeepView Tool [40] was used to align sequences of known structure, then apoptin sequence was threaded to the crystal structure of PDB id 1WLS and then submitter for model building. Modeller [38], [39] a web-based server was also used in model building. Several other computer programs [42], [43], [44], [66] were used to build and process the apoptin model using 121 amino acids sequence. Several models building were performed using different templates and accuracy was examined. One of the best models was used for further studies. All other calculations including molecular dynamic simulation and visualization of 3D structure were performed using Scigress Explorer Ultra [46]. After building the 3D model of apoptin, all atomic positions are locked and required hydrogen atoms were added to the backbone structure of the apoptin molecule and performed ‘molecular mechanics’ calculations and then performed molecular dynamics simulation at 1000 k for 50 ps for further optimization. One of the best apoptin models was used to examine the solvent accessible surface area. A docking file with pdb extension of apoptin molecule was prepared without hydrogen atom to perform molecular docking p17experiments to examine the interaction between apoptin model and the Bcr-Abl oncoprotein using 3D structure of the protein (PDB code: 2ABL).

### Validation of 3D Model

After building the 3D apoptin model, the Protein Structure & Model Assessment Tools [42], [43], [44] was used to verify the quality of the apoptin model. This tools is capable of verifying a number of aspects of model qualities such as (1) Local Model Quality Estimation (anolea atomic mean force potential, empirical force field, composite scoring function for model quality estimation); (2) Global Model Quality Estimation (all-atom distance-dependent statistical potential); (3) Stereochemistry Check (protein structure verification, stereochemical quality check; min. resolution 2.5 Å) and (4) Structural Features (secondary structure, geometrical features, and solvent exposure assignment, analysis of protein structure motifs). Based on these assessment results, model quality was evaluated according to the Ramachandran plot and the amino acid residues in the allowed, disallowed region and overall G- factor.

### Molecular Docking of Apoptin model to the 3D Structure of SH3 domain of Bcr-Abl

Three (Hex, ClusPro, and AutoDock) computer programs [45], [48] were used to perform the docking experiments. All docking programs required two separate input files for the two molecules, apoptin model and 3D structure of SH3 domain of Bcr-Abl, without hydrogen atoms with pdb extension. The ClusPro provides greater and accurate information. Several apoptin models were used to perform docking experiments using ClusPro docking server. After the docking experiments, the ClusPro server provided about 10 structures. The lowest energy docking structure was used for further studies. After the docking experiments, all other modeling and calculations were performed using DeepView and Scigress Explorer Ultra. All atomic positions of the lowest energy docking structures are locked and required hydrogen atoms were added to the structures and performed ‘molecular mechanics’ calculations to minimize the energy for the added hydrogen atoms. This minimize structure was used to examine and to identify the interacting residues between apoptin and the Bcr-Abl molecules. Hydrogen bonding of the interacting residues between the two proteins was also examined. These molecular interactions were further verified biochemically.

### Pathway, Interacting Network using and Global Gene Expression data

Pathways and gene/protein interacting networks were examined from a global prospective using bioinformatics tools and microarray gene expression data, such as ‘GenMapp’ and ‘Ingenuity Pathway Analysis’. We used the publicly available gene expression data from K562, a leukemia cell line expressing Bcr-Abl. Two input files specific for each bioinformatics tool were prepared. Expression of some of important molecules was validated experimentally in the presence and absence of apoptin.

### Cell lines, plasmids, Cell death and cell proliferation assays, antibodies and reagents

All cell culture media and supplements were from Gibco BRL. 32D^DSMZ^ and 32D^p210^wt:b3:a2/e13:a (denoted as 32D^p210^) [67] Bcr-Abl variant were grown in RPMI-1640 cell culture medium 500 ml supplemented with 20% FBS (Hyclone), 0.05 g penicillin/streptomycin, 10 mM HEPES (2-(4-hydroxyethyle)-1-piperazinyl)ethanolsulfonsäure), 2 mM L-Glutamine, 0.13 mM L-Asparagin, 0.05 nM 2-Mercaptoethanol, 1 mM Na-Pyruvate, and 3 ml 100× non-essential amino acids. 32D_DSMZ_ cells are strictly dependent on murine interleukin 3, so the media was supplemented with 10% supernatant from WEHI-3B cells [67]. The human CML cell line K562 (ATCC® # CCL-243™) was cultured in ATCC recommended Iscove's modified Dulbecco's medium with 4 mM L-glutamine adjusted to contain 1.5 g/L sodium bicarbonate, 10% FCS (v/v) and antibiotics [68], [69]. All cells were grown at 37°C with 5% CO_2_ in a humidified incubator and maintained in a logarithmic growth. The following plasmids were used: GFP, GFP-Apoptin (apoptin cloned into pEGFP-C1 vector, Clontech), GST, GST-Apoptin (apoptin cloned into PGEX-2T vector, Amersham Biosciences), apoptin mutants were previously described [19]. The p210Bcr-Abl mutants (kind gift from Dr. T. Skorski, Temple University, USA), ΔSH2 (deletion of the SH2 domain), ΔSH3+ΔSH2 (deletion of both the SH3 and the SH2 domains), ΔSH3+R1053L (deletion of the SH3 domain and single aa substitution in the SH2 domain) and P1013L+R1053L (single aa substitutions in the SH3 and SH2 domains), cloned in pSRaMSVtkneo vector as described [70]. Plasmids were propagated either in BL21(DE3)pLysS or DH5α *E. Coli* strains, upon transformation by CaCl_2_ chemical method. Plasmid isolation (positive clones) was done using Qiagen Maxi-prep kits. Cell proliferation was assessed by MTT assay, and cell death was measured by propidium iodide uptake as previously described [71].

Chemicals and antibodies were purchased from Sigma-Aldrich® Inc. (Sigma, Oakville, ON, Canada) Abcam® Inc. (Cambridge, MA, USA) or Cell Signaling Technology®, Inc. (Danvers, MA, USA). The following antibodies were used: murine/rabbit anti-Bcr-Abl/anti-Bcr (monoclonal: Abcam® Inc.), murine anti-Akt (monoclonal) and the murine monoclonal anti-apoptin antibody (kind gift from Dr. D. Jans, Australia).

### Transfection of mammalian cells

Different mammalian primary and cancer cell lines were transfected with the desired plasmids by Lipofectamine™2000 (Invirogen® Canada Inc. Burlington, Ontario, L7P 1A1) reagent. The cells were plated in an antibiotic free medium 24 h prior to transfection and plasmid DNA was added to 100 µl of medium in a tube at the recommended concentrations (typically 2 µg for a 12 well plate and 5 µg for a 6 well plate) at the time of transfection. The Lipofectamine reagent (5 µl for 12 well and 10 µl for 6-well plate) was diluted in 100 µl of medium in a second tube. Next, the DNA and Lipofectamine reagents were mixed after 5 min and then incubated for 20 min at room temperature to allow the formation of DNA liposome complexes. Following the incubation, the DNA-lipid mixture was gently added directly to the cells that had previously been rinsed with PBS and replaced with fresh medium.

### GST-pull down assay and protein identification, co-immunoprecipitation (Co-IP), Western blotting

The GST and the recombinant GST-apoptin proteins were purified according to the manufacturer's protocol using glutathione sepharose beads (Amersham Biosciences®). GST-pull down assay was performed to detect the interacting partners of apoptin. Briefly, either purified GST or GST-apoptin along with total 32D^p210^ or K562 cell lysate (by sonification) were immobilized on glutathione sepharose beads overnight at 4°C in IP buffer with protease and phosphatase inhibitors (50 mM Tris-HCl pH 8.0, NaCl 150 mM, ND-40 0.5%, EDTA 1 mM, PMSF 1 mM, NaF 10 mM, Na_3_VO_4_ 1 mM, β-glycerophosphate 25 mM). Beads were washed at least six times with ice-cold lyses buffer and the bound proteins were detected by Western blotting.

For Co-IP experiments, 2–5 µg of antibody was added to 100–500 µg of cell lysate and incubated (4 hr at 4°C) and then 100 µl of equilibrated 50% protein-G Sepharose beads (Amersham Pharmacia Biotech®) were added to the protein-antibody immune complexes and incubated (1 h at 4°C). Beads were washed (6×) with lyses buffer and after the final wash, beads were suspended in SDS sample buffer and resolved on SDS-PAGE gel, and the Co-IPed protein was detected by Western blotting. Other Western blotting experiments were performed as previously reported [72], [73], [74]. Briefly, about 30 µg protein lysates were resolved by SDS PAGE, transferred to PVDF-membrane (Amersham Biosciences®), membrane blocked (5% non-fat dry milk powder/tris-buffered saline with 0.25% v/v Tween-20 5% bovine serum albumin, BSA). Membranes were washed and incubated with an appropriate secondary antibody conjugated with horseradish peroxidase (HRP) for 45 min at room temp and detected by using enhanced chemiluminescent (ECL) staining (Amersham Biosciences®).

### Immunocytochemistry and fluorescent imaging

Cells were allowed to attach overnight, transfected with appropriate plasmids and after 16–18 h of incubation cells were collected, washed with PBS, and fixed in 4% w/v paraformaldehyde/PBS. Thin smear of cells was prepared on standard microscope glass slides and air-dried. Cells were permeabilized (0.1% triton X-100/PBS), blocked (5% BSA/PBS, 1 h) and incubated overnight at 4°C with an appropriate primary antibody, followed by incubation with Cy3 or FITC conjugated secondary antibody. Slides were mounted with Vectashield® containing DAPI. Fluorescence signals were acquired using Zeiss fluorescent microscope and analyzed by Zeiss Axiovision (Version 3.1) software.

### Bcr-Abl multiplex kinase assays

The phosphorylation status of Bcr-Abl, STAT5, CrkL and Akt were measured by scanning the signal strength of phosphorylated proteins on Western blot membranes. Kinase reaction was performed in K562 and 32D^p210^ cells by overnight (16 h) incubation with Tat-apoptin (test), Imatinib® (positive control) Tat-GFP, or no treatment (negative controls) and cell extracts were resolved by SDS-PAGE (10%) and detected by immunoblotting using respective phospho-specific antibodies or antibodies detecting both phosphorylated and non-phosphorylated kinase. A high-resolution scanner (STORM 860: Molecular Dynamics®: Amersham Pharmacia Biotech) was used to scan the immunoblot membranes after ECL detection and individual band intensities were measured by Image Quant (Version 5.2) software (Molecular Dynamics®). Band intensities were normalized to the respective loading controls and expressed as a ratio of measured values for phosphorylated proteins versus total protein bands.

### Statistical Analysis

Unless stated otherwise, all normalized band intensity data were statistically analyzed by *student's t test assuming equal variance* using Microsoft® Excel software. The variance patterns in each set of data were previously checked by ANOVA from Excel data analysis tool package.

## Supporting Information

Coordinate S1 **Apoptin structure coordinates.**
(TXT)

Coordinate S2 **Apoptin structure coordinates.**
(TXT)

Coordinate S3 **Apoptin structure coordinates.**
(TXT)

Coordinate S4 **Coordinates of Apoptin docking to BcrAbl.**
(TXT)

Coordinate S5 **Coordinates of Apoptin docking to BcrAbl.**
(TXT)

Coordinate S6 **Coordinates of Apoptin docking to BcrAbl.**
(TXT)
